# Modelling the spatial risk of malaria through probability distribution of *Anopheles maculipennis* s.l. and imported cases

**DOI:** 10.1080/22221751.2024.2343911

**Published:** 2024-04-15

**Authors:** Shirin Taheri, Mikel Alexander González, María José Ruiz-López, Sergio Magallanes, Sarah Delacour-Estrella, Javier Lucientes, Rubén Bueno-Marí, Josué Martínez-de la Puente, Daniel Bravo-Barriga, Eva Frontera, Alejandro Polina, Yasmina Martinez-Barciela, José Manuel Pereira, Josefina Garrido, Carles Aranda, Alfonso Marzal, Ignacio Ruiz-Arrondo, José Antonio Oteo, Martina Ferraguti, Rafael Gutíerrez-López, Rosa Estrada, Miguel Ángel Miranda, Carlos Barceló, Rodrigo Morchón, Tomas Montalvo, Laura Gangoso, Fátima Goiri, Ana L. García-Pérez, Santiago Ruiz, Beatriz Fernandez-Martinez, Diana Gómez-Barroso, Jordi Figuerola

**Affiliations:** a Departamento de Biología de la Conservación y Cambio Global, Estación Biológica de Doñana (EBD), CSIC, Sevilla, Spain; b CIBER de Epidemiología y Salud Pública (CIBERESP), Madrid, Spain; c The Agrifood Institute of Aragón (IA2), Faculty of Veterinary Medicine, University of Zaragoza, Zaragoza, Spain; d Center of Excellence in Vector Control, Rentokil Initial, València, Spain; e Grupo de Investigación Parásitos y Salud, Universitat de València, València, Spain; f Departamento de Parasitología, Universidad de Granada, Granada, Spain; g Departamento de Salud Animal, Grupo de Investigación en Salud Animal y Zoonosis (GISAZ), Facultad de Veterinaria, Universidad de Córdoba, Córdoba, Spain; h Departamento de Sanidad Animal, Facultad de Veterinaria, Universidad de Extremadura (UEx), Cáceres, Spain; i Departamento de Ecoloxía e Bioloxía Animal, Universidade de Vigo, Pontevedra, Spain; j Departamento de Zooloxía, Xenética e Antropoloxía Física, Universidade de Santiago de Compostela, A Coruña, Spain; k Servei de Control de Mosquits del Baix Llobregat, Sant Feliu del Llobregat, Barcelona, Spain; l IRTA, Centre de Recerca en Sanitat Animal (CReSA, IRTA-UAB), Campus Universitat Autònoma de Barcelona, Bellaterra, Spain; m Facultad de Biología, Universidad de Extremadura, Badajoz, Spain; n Grupo de Investigaciones en Fauna Silvestre, Universidad Nacional de San Martín, Tarapoto, Perú; o Centre of Rickettsiosis and Arthropod-Borne Diseases, Hospital Universitario San Pedro-CIBIR, La Rioja, Logroño, Spain; p Centro Nacional de Microbiología (CNM-ISCIII), Madrid, Spain; q CIBER de Enfermedades Infecciosas (CIBERINFEC), Madrid, Spain; r Universitat de les Illes Balears (UIB), Zoología Aplicada y de la Conservación, Palma, Spain; s Zoonotic Diseases and One Health Group, Faculty of Pharmacy, Universidad de Salamanca, Salamanca, Spain; t Agencia de Salut Publica de Barcelona, Barcelona, Spain; u Universidad Complutense de Madrid, Madrid, Spain; v NEIKER-Instituto Vasco de Investigación y Desarrollo Agrario, Derio, Spain; w Servicio de Control de Mosquitos de la Diputación de Huelva, Huelva, Spain; x Centro Nacional de Epidemiologia (CNE-ISCIII), Madrid, Spain

**Keywords:** Paludism, pathogeography, spatial epidemiology, species distribution modelling, risk maps, vector-borne diseases

## Abstract

Malaria remains one of the most important infectious diseases globally due to its high incidence and mortality rates. The influx of infected cases from endemic to non-endemic malaria regions like Europe has resulted in a public health concern over sporadic local outbreaks. This is facilitated by the continued presence of competent *Anopheles* vectors in non-endemic countries.

We modelled the potential distribution of the main malaria vector across Spain using the ensemble of eight modelling techniques based on environmental parameters and the *Anopheles maculipennis* s.l. presence/absence data collected from 2000 to 2020. We then combined this map with the number of imported malaria cases in each municipality to detect the geographic hot spots with a higher risk of local malaria transmission.

The malaria vector occurred preferentially in irrigated lands characterized by warm climate conditions and moderate annual precipitation. Some areas surrounding irrigated lands in northern Spain (e.g. Zaragoza, Logroño), mainland areas (e.g. Madrid, Toledo) and in the South (e.g. Huelva), presented a significant likelihood of *A. maculipennis* s.l. occurrence, with a large overlap with the presence of imported cases of malaria.

While the risk of malaria re-emergence in Spain is low, it is not evenly distributed throughout the country. The four recorded local cases of mosquito-borne transmission occurred in areas with a high overlap of imported cases and mosquito presence. Integrating mosquito distribution with human incidence cases provides an effective tool for the quantification of large-scale geographic variation in transmission risk and pinpointing priority areas for targeted surveillance and prevention.

## Main

Among vector-borne diseases, malaria remains one of the most serious infectious diseases with an estimated 247 million infected human cases and 619,000 deaths in 2021 (most occurring in Africa) [1]. On the positive side, malaria was eradicated from many countries, including the USA and Canada by the 50s and Europe by the 70s. However, the competent malaria vectors (many species of genus *Anopheles*) are still present in these non-endemic regions. A recent study estimated that approximately 50,000 infected people travelled between 2005 and 2015 from malaria-endemic areas to 40 non-endemic countries [2]. In the European Economic Area between 8,200 and 8,600 imported malaria cases were reported annually for the period 2015–2017 [3]. As a result, local transmission cases are sporadically recorded in many non-endemic territories, including Europe. Most of these imported cases are attributed to *Plasmodium falciparum* (81.8%) but also include infections by *Plasmodium vivax* (4.9%) and *Plasmodium ovale* (2.7%) [3]. An important outbreak of *P. vivax* was reported in Southern Greece in 2011–2012, where local transmission occurred for several consecutive years [4]. Among the vast number of approximately 530 *Anopheles* species that exist in the world, only 30–40 species are capable to transmit malaria in nature [5].

Mosquito abundance and distribution are largely determined by climate and landscape [6,7]. Considering multifaceted factors, including climate, environmental conditions, vector presence, number of imported cases and disease dynamics are crucial for predicting regions that are susceptible to future outbreaks [8,9]. For example, understanding and quantifying where a high incidence of imported malaria cases spatially overlap with a high habitat suitability of vectors will improve our ability to prioritize areas for mosquito control and disease prevention in non-endemic regions. Although malaria in Spain was officially eradicated in 1964 [10], four cases of vectorial transmission, and 28 other cases of non-vectorial transmissions, such as congenital, transfusion, nosocomial, or transmissions between intravenous drug users have been recorded in the following years [10]. This highlights the need to investigate the presence of *Anopheles* vectors that are competent in those areas where imported cases have already been detected.

Despite significant efforts to develop risk maps for vector-borne diseases, there remains a lack of comprehensive studies that fully integrate multiple factors into these maps. Existing research often focuses on specific aspects, such as vector distribution and expansion [11–13], disease prevalence [14], and imported cases [15–17]. While these factors are individually important, it is the interaction between them that would ultimately determine the risk of transmission of imported cases into local populations. Unfortunately, detailed information on vector distribution is often geographically biased and scarce. Moreover, although much of this information has been collected in the recent years, it remains uncounted in large biodiversity databases (e.g. GBIF (Global Biodiversity Information Facility)).

To fill this gap, we integrated habitat suitability models for the main *Anopheles* vector of *Plasmodium* present in Spain with data on the distribution of human-imported cases from the RENAVE (Spanish National Epidemiological Surveillance Network). We identified the areas with a high relative risk of local malaria transmission through the combined effects of malaria vector suitability and the distribution of imported cases.

## Material & methods

- Entomological data collection

The *A. maculipennis* complex includes the main malaria vectors present in Europe [18]. The data from the most prevalent species (*A. maculipennis* s.l.) were obtained from various sources, including nine Spanish research groups, national mosquito surveillance centres, and public health control agencies. The data encompassed unpublished and regional monitoring programme data from large-scale projects such as the Bluetongue National Surveillance Program and REGAVIVEC from the Galician Network of Vector Surveillance, complemented with data from international databases like GBIF and iNaturalist, and published sources. The sampling includes a total of 5749 records between 2000 and 2020, for all Spanish provinces except for those of the Canary Islands, which have very different climatic patterns and were thus excluded from our analysis.

- Molecular identification of the *Anopheles maculipennis* complex

To estimate the frequency of the different species of the *A. maculipennis* complex across Spain, we conducted a literature review and a molecular identification of 121 adult specimens. These mosquitos were sampled from 2019 to 2023 in 10 Spanish regions (Figure S1) and preserved either in 70% ethanol or stored at −80°C until further analysis. We extracted genomic DNA from single mosquitoes using the Maxwell® 16 LEV system Research (Promega, Madison, WI) with the Maxwell ® 16 LEV Blood and Tissue DNA kit, following the manufacturer's protocol. Molecular identification of the sibling species of the *A. maculipennis* complex was carried out using a PCR-RFLP protocol [19], (see Supplementary material for further details of the procedure and the information gathered from the literature).

- Human imported cases

Data on the imported cases of malaria between 2005 and 2020 were obtained from RENAVE. The data provide information for the municipality where the case was registered, the residence municipality, the country of residency, the country of origin, and the date of diagnosis for each patient. For the analyses, we only considered records with information on the residence municipality (5718 out of 8254 records). The polygon shapefiles of the municipal boundaries of Spain were obtained from Eurostat (https://ec.europa.eu/eurostat).

To effectively visualize the spatial distribution of imported cases and overlap them with the probability distribution of the vector, we used municipality codes as a unique code to join the number of imported malaria cases in each municipality to the vector distribution map.

## Vector distribution and environmental parameters

To understand the association between climate variables and *A. maculipennis* s.l. distribution*,* we used monthly time series of climate data from the Terraclimate dataset [20]. The dataset provides climate information on minimum and maximum temperature and precipitation with a spatial resolution of approximately 4 km. We particularly focused on a period from 2000 to 2020, aligning with the availability of the species points. We then used this information to drive the corresponding set of bioclimatic variables using the R package *dismo* [21].

**Figure 1.**
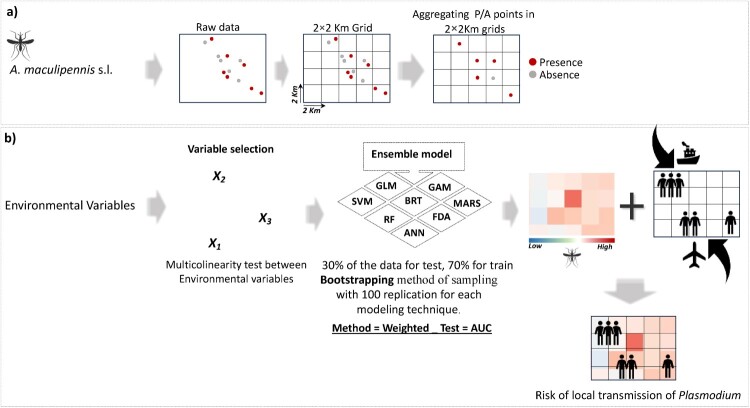
General methodological framework. The overall process for identifying high-risk transmission areas according to the high probability distribution of the vector and the incidence of imported malaria. **a)** Data preparation: this step involves cleaning and wrangling mosquito occurrence points to ensure data quality and consistency. **b)** Modelling techniques: details used to identify areas with a high probability of *A. maculipennis* s.l. occurrence and the number of imported cases in each Spanish municipality.

We expanded our analysis by incorporating wind speed and runoff data into our model, which were sourced from the same data provider, with the assumption that factors such as high wind speed and runoff could affect mosquitoes’ distribution and survival. Our choice of variables reflects those known to impose general constraints on the suitability of *A. maculipennis* s.l. distribution (See Table S1) [22,23]. Moreover, to avoid issues related to spatial autocorrelation [24,25], we generated regular grids with 2 × 2 km cell size and assigned presence/absence points to the corresponding grid cell, ensuring that only one point lies inside each grid cell (Figure 1a). *Anopheles maculipennis* s.l. was considered present when at least one sample point inside the grid cell indicated its presence and was considered absent when all the points inside the cell were absences. In this way, we removed duplicated points and those that had nearest neighbours at a distance lower than 2 km (Figure S1).

We downscaled the climate data using Inverse Distance Weighting (IDW) following the approach proposed by Donald Shepard, (1967) [26] and using the *raster* package in R [27]. IDW is a method of interpolation that estimates cell values by averaging the values of sample data points in the neighbourhood of each processing cell. The closer a point is to the centre of the cell being estimated, the more influence, or weight it has in the averaging process [28]. The output cell size and other parameters of the new raster layers were matched to the 2 × 2 km grid of the *A. maculipennis* s.l*.* occurrence points (Figure 1a).

To avoid multicollinearity, we calculated the Variance Inflation Factor (VIF) and absolute correlation coefficient between all predictor variables using the *rsdm* R package [29]. Variables with VIF > 10 were excluded from the models. As a result of variable selection and multicollinearity test, we ended up with the following variables: annual mean temperature (bio.1), temperature seasonality (bio.4), max temperature of the warmest month (bio.5), annual precipitation (bio.12), precipitation seasonality (bio.15), annual runoff, and wind speed.

We obtained CORINE-land-cover 2018 from Copernicus (European Union’s Earth observation programme). The CORINE dataset is available in shapefile format with 44 classes. To reduce the complexity of the analysis, we reclassified original categories into seven land cover classes: urban area, urban green space, irrigated lands, agricultural lands, inland waters (water bodies), open spaces (e.g. beaches, dunes, sands, bare rocks, burnt areas, glaciers, and perpetual snow), and natural ecosystems (e.g. broad-leaved forest, coniferous forest, mixed-forest, natural grasslands, moors and heartland, sclerophyllous vegetation, transitional woodland-shrub, sparsely vegetated areas). The proportion of each of the seven land-cover categories was calculated within each 2 × 2 km grid of species ranges. All the analyses were carried out in RStudio version 3.3.1 (R Core Team 2019).

- Statistical analysis

To examine the association between climate and land-cover prediction with the presence/absence of *A. maculipennis* s.l*.,* we developed an ensemble species distribution model using the *sdm* package in R [29]. We modelled the presence/absence distribution of the vector as a function of the six climate and seven land-cover variables. To improve the robustness of our model, we used an ensemble modelling framework [30] integrating eight different algorithms for modelling the presence/absence of *A. maculipennis* s.l*.*: generalized linear models (GLM; [31]), generalized additive models (GAM; [32]), random forest (RF; [33]), flexible discriminating analysis (FDA; [34]), boosted regression tree (BRT; [35]), support vector machines (SVM; [36]), multivariate adaptive regression splines (MARS;[35]), and artificial neural network (ANN; [37]) (Figure 1b). Ensemble modelling helps to address uncertainties associated with model selection and variability by incorporating multiple models with different algorithms, data, and assumptions [30]. It also improves prediction accuracy and provides more robust and reliable insights, reducing the impact of individual model limitation error (see also [38]). The variability in variable importance across different modelling techniques stems from differences in model characteristics, assumptions, how they handle and interpret the data, and the modellings of variable interactions (Figure S2). That is why it is highly recommended to use ensemble models, which combine multiple models and generally offer more reliable variable importance measures [38].

To avoid biases in parameter estimation, we used a bootstrapping method [39,40] with 100 random replications for each modelling technique. Bootstrapping repeats a sampling with replacement, each time drawing a sample with equal size as the original data for training data. The observations that are not selected are used for the evaluation at each run. We then generated a consensus model, using the weighted average probability for vector data, where the weight was obtained from the area under the curve (AUC) in evaluation data (e.g. [41]).

To identify high-risk areas for malaria, we first computed the incidence rate in each municipality by dividing the number of positive imported cases by the population living in the municipality [42]. Second, we multiplied the number of incidences by the median probability distribution of the vector species within each municipality to find the hot-spot areas where the high probability of the vector coincides with a high malaria incidence rate.

## Results

The joined data of the molecular analyses and literature review showed that 78,6% of the total individuals analyzed in Spain (n = 229) were *A. atroparvus*. This species was predominant in all the regions analyzed. The remaining 21,4% (n = 49), identified as *A. maculipennis* s.s., were mainly found in the northern areas of Spain, co-occurring with *A. atroparvus* (Figure S3). Most *A. maculipennis* s.s. occurred in País Vasco (46 of the 50 individuals identified as *A. maculipennis* s.s. for all of Spain) and outside north Spain, *A. atroparvus* represented 97,3% of the analyzed individuals. Consequently, *A. atroparvus* can be considered the most common species within the *A. maculipennis* complex in Spain.

**Figure 2.**
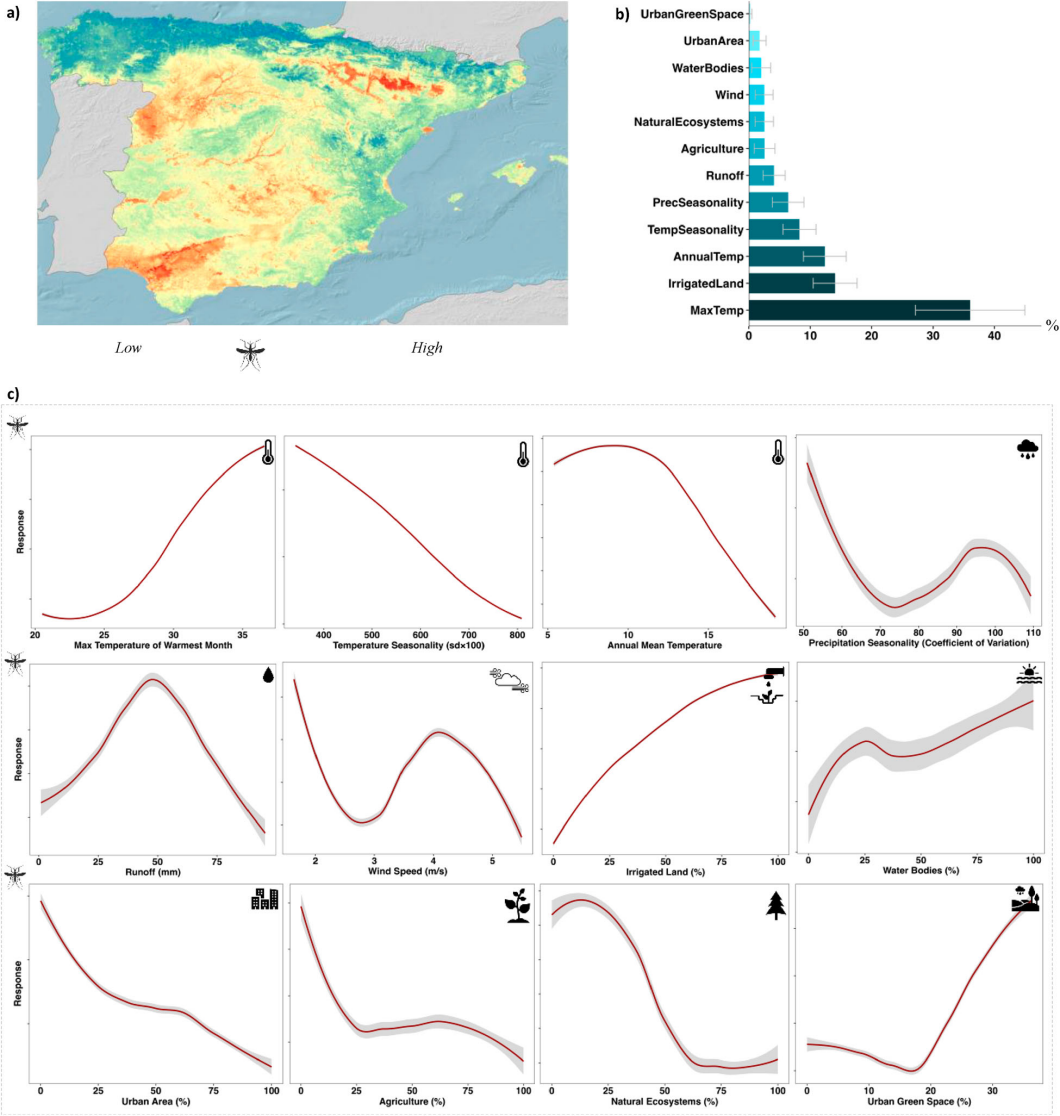
Habitat suitability, variable importance, and response curves of *A. maculipennis* s.l. using an ensemble of eight modelling techniques and 100 repetitions for each model. **a)** Habitat suitability, **b)** Variable importance (% IncMSE): quantifies the relative contribution of 12 environmental variables in modelling the habitat suitability of the vector (darker colours correspond to the higher contribution of the model), **c)** Response curves illustrate the relationship between the probability of occurrence of A. maculipennis s.l. (response) in the Y-axis and the corresponding environmental variables (X-axis). The 95% confidence level interval is shown in grey.

The ensemble of eight modelling techniques clearly showed that *A. maculipennis* s.l. has a strong inclination towards irrigated lands (e.g. rice fields and permanently irrigated lands) in areas with warm climate (Figure 2b). Overall, habitat suitability for *A. maculipennis* s.l. increased as the maximum temperature in the warmest month increased (Figure 2c) and decreased with high-temperature seasonality (the amplitude of annual cycle temperature) and mean annual temperature.

Moderate precipitation increased the likelihood of *A. maculipennis* s.l. occurrence, although high levels of precipitation decreased its suitability. The same trend was observed for wind speed and surface runoff water, where moderate levels of these variables positively influenced the suitability for *A. maculipennis* s.l., while higher levels had a negative impact (Figure 2c). However, these two variables make a small contribution to the model fit (Figure 2b). These findings revealed that the suitability of the vector decreased in areas characterized by intensive agricultural lands where more than 40% of the land within a 2 km grid was dedicated to crop cultivation. Furthermore, natural ecosystems, such as broad-leaved forests, coniferous forests, natural grasslands, and heathlands, were also found to contribute to the association reducing the suitability of the vector habitat. In addition, while urban areas typically exhibit lower suitability for *A. maculipennis* s.l*.* mosquitoes, the presence of green urban spaces and sport leisure facilities within cities can create microhabitats that are conducive to the presence of vectors and reproduction.

**Figure 3.**
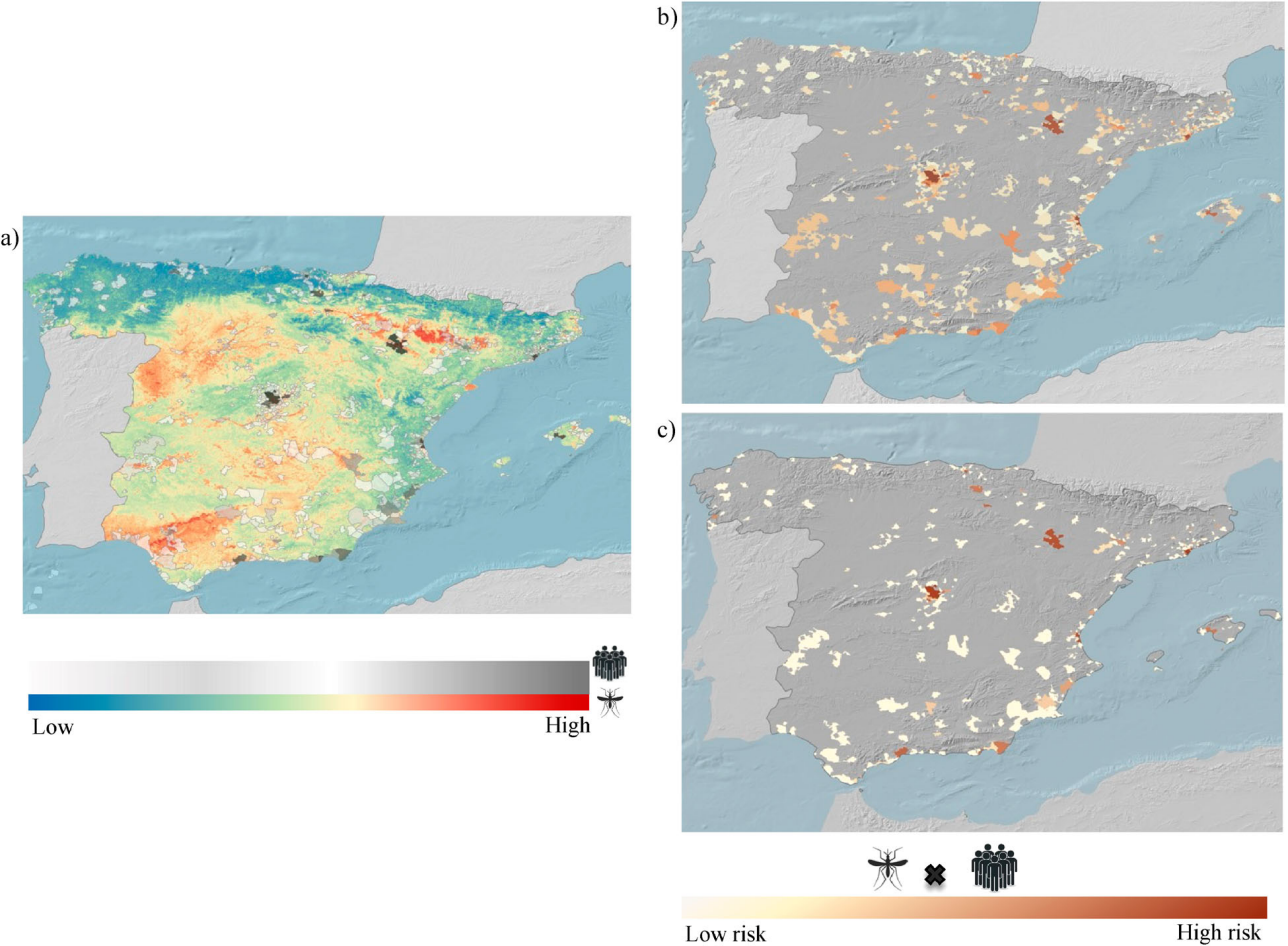
The spatial distribution of transmission risks associated with the high probability distribution of *A. maculipennis* s.l. and imported malaria cases. **a)** The spatial overlap between imported cases and the probability distribution of *A. maculipennis* s.l. **b)** The spatial overlap between the probability distribution of *A. maculipennis* s.l. and malaria incidence rate (the ratio of confirmed cases of malaria divided by the total population in each municipality). **c)** The risk areas of malaria after excluding *P. falciparum* from the imported cases.

The primary locations for the presence of *A. maculipennis* s.l. are in central Spain (e.g. Madrid, Toledo) as well as areas surrounding the main rivers and associated irrigated lands in the North-East and South-West of Spain. These areas include the Ebro River valley (e.g. Zaragoza, Huesca), the Guadalquivir River valley and nearby agricultural areas (e.g. Cordoba, Huelva) (Figure 3).

Overall, our models demonstrated good modelling performance with an AUC ranging between 0.7 and 0.8 for all modelling techniques, except RF which had a very good performance <0.9 (Figure S4 and Figure S5). RF outperforms other methods due to its ensemble learning nature, aggregating predictions from multiple decision trees via bagging (bootstrap aggregation), which prevents overfitting by combining predictions from diverse trees trained on varied data subsets.

**Figure 4.**
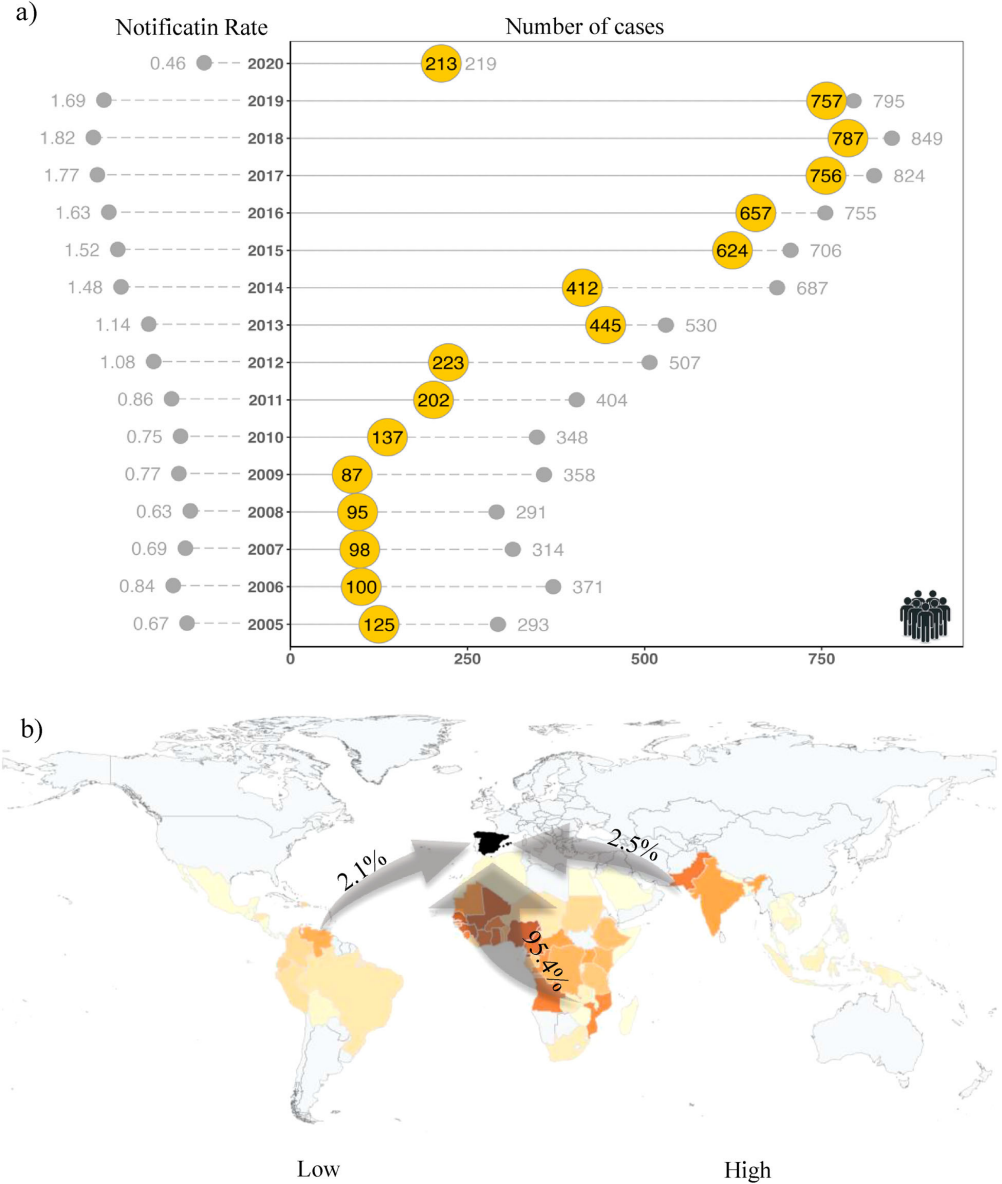
Number and origin of malaria cases in Spain. **a)** Indicates the general trend of imported malaria cases from 2005 to 2020. The dashed line represents the total number of imported malaria cases, while the solid line represents the subset of imported cases with registered municipalities. The dashed line on the left side represents notification rate (reported cases in relation to the total population ×100,000). **b)** Geographic distribution and country of origin of imported malaria cases in Spain.

Between 2005 and 2020, a total of 8254 imported malaria cases were identified in Spain. Most of them were produced by *P. falciparum* (n = 6795, 82,3%), with smaller numbers of cases attributed to *P. vivax* (n = 352, 4,3%), *P. ovale* (n = 191, 2,3%), and *P. malariae* (n = 132, 1,6%). The *Plasmodium* species was not reported for 625 cases (7.7%, Figure S6, Figure S7). The major sources of imported malaria cases in Spain can be traced back to various countries in sub-Saharan Africa, such as Equatorial Guinea (n = 1523), Nigeria (n = 852), Mali (n = 781), Senegal (n = 451), Guinea (n = 291), Ghana (n = 276), and Cameron (206). Additionally, South American countries, including Venezuela (n = 25), Ecuador (n = 15), Colombia (n = 14), and Honduras (n = 17), as well as certain countries in South and Southeast Asia, such as Pakistan (n = 87), India (n = 28), Cambodia (n = 14), and Indonesia (n = 5) have also contributed to the imported malaria cases observed in Spain during the study period (Figure 4b). The number of imported malaria cases in Spain follows an upward trend over time rising from 293 in 2005 to 849 and 795 in 2018 and 2019, respectively (Figure 4a). However, in 2020, there was a substantial decline to 213 cases. In addition, the number of reported cases varied by region, with more cases identified in Madrid (n = 1187), Cataluña (n = 1198), Comunidad Valenciana (n = 664), País Vasco (n = 476), and Aragón (n = 293; 76.4% of cases in Zaragoza). In certain instances, these areas coincided with regions highly suitable for *A. maculipennis* s.l. (Figure 3a), resulting in a more extensive overlap between vectors and imported cases in irrigated lands in the Southeast of Spain, Aragón, and Madrid. Notably, *A. maculipennis* s.l. is present in neighbourhoods of the South of Madrid overlapping with an important number of imported cases (Figure 3b,3c).

## Discussion

Here, we identified potential hotspots for malaria transmission combining the information of imported malaria cases with a detailed analyses of the environmental factors that determine the presence of *A. maculipennis* s.l. Our models suggest that the combination of high summer temperature with low-temperature variability, moderate precipitation and the presence of irrigated lands create a suitable environment for the proliferation of *A. maculipennis* s.l. [40].

Hence, Spanish municipalities located near irrigated lands with warm summer temperatures are likely to hold significant populations of *A. maculipennis* s.l.. These species exhibit a pronounced preference for the Continental climatic zones in Spain, as it is predominantly found in these specific regions (Figure 2a). Seemingly, the Continental Mediterranean climate, characterized by distinct seasonal dynamics encompassing hot summers and cold winters, along with regions featuring the Mediterranean climate with hot and dry summers, provide suitable environmental conditions for the distribution and proliferation of *A. maculipennis* s.l..

The overlap between mosquito distribution maps with the imported malaria cases indicates the presence of high-risk areas associated with irrigated lands (e.g. rice fields) in different parts of Spain (e.g. the Ebro valley and Huelva). Additionally, different localities around Madrid also present a large degree of overlap between imported cases and habitat suitability for *Anopheles* mosquitoes. Notably, in 2001, a locally acquired case of malaria produced by *P. ovale* was detected in a person who had not travelled to endemic countries in a locality of East of Madrid [43]. Also, a previous case of infection by *P. falciparum* in a nearby locality was reported in 1984 [44]. While these two cases may be considered cases of airport malaria, i.e. caused by the arrival of infected vectors because of airport activities, the distance of these localities to the airport and the activity patterns of the two infected persons could also be compatible with transmission by locally infected vectors. Afterwards, in 2010, another infection by *P. vivax* was detected in a resident in a locality close to the Ebro valley in Huesca, with the onset of symptoms in September [45]. Four years later, another infection by *P. vivax* genetically related to a nearby imported case from Pakistan, was reported in a locality of Navarra, starting symptoms in August [46]. These two cases highlight how the distribution of the sporadic cases of local malaria transmission in rural or nearby areas of Huesca and Navarra overlaps with the high-risk areas highlighted in our study (Figure S8), with the season of maximum suitability (e.g. temperature, humidity) for local vectors to be active. At the moment, the risk of local transmission of malaria imported from Africa in Spain is considered low, bearing in mind that *A. atroparvus* is competent for *P. vivax* but seems to be refractory to *P. falciparum* tropical strains, which are responsible for the majority of imported cases [47]. However, the risk may be higher in regions with a high suitability for vectors and an increased likelihood of imported cases originating from endemic regions where *P. vivax* circulates, such as Latin America or Asia. The exclusion of *P. falciparum* from our analysis underscores the comparability, as it still identifies the same high-risk regions (Figure 3b,3c).

Moreover, a majority of the imported cases tend to occur during months characterized by favourable conditions for the development of *Anopheles* mosquitoes and temperature conductive to *Plasmodium* transmission (see Figure S9). Considering the above-mentioned reasons along with the possibility of ongoing changes in travel patterns and in the malaria epidemiology in endemic countries, we should be aware about the need of updating risk assessment based on all these factors and incorporate surveillance of *Anopheles* mosquitoes to the National Plan for the prevention, surveillance, and control of vector borne pathogens [48].

However, this should be considered as a relative indicator of transmission across Spain, and not as an absolute estimator, because the low incidence of autochthonous transmission suggests that establishment risk remains low. The widespread distribution of *A. atroparvus* highlights the need for early detection and treatment of malaria imported cases. Moreover, the risk maps we provided here aim to guide public health strategies and interventions by allowing stakeholders to prioritize resources and implement targeted mosquito prevention and control measures in the areas where the potential for disease transmission is higher [49]. In these areas, favouring the use of personal protection with topical repellents and other mosquito prevention measures, may successfully reduce disturbance by *Anopheles* mosquitoes and the risk of transmission of mosquito-borne pathogens.

Accurately estimating the risk areas associated with the habitat suitability of *Anopheles* mosquitoes requires robust data and advanced methodologies, encompassing predictor selection and modelling techniques. In this study, we used a unique dataset containing the presence/absence of *A. maculipennis* s.l., which was collected in collaboration with numerous entomologists throughout Spain from 2000 to 2020. Nevertheless, it is important to acknowledge that this dataset may be subjected to certain data uncertainties and biases. For example, we are aware that a small percentage of the records may correspond to any of the two sibling species of the *A. maculipennis* complex, although our molecular analyses support that *A. atroparvus* may dominate the captures included here. Similar data may exist for many other regions in Europe, but up to now, it is not easily available through biodiversity platforms such as GBIF. Therefore, the collaboration between field ecologists, entomologists, modellers, epidemiologists, and public health services is necessary to reverse this situation.

However, while the geographic distribution of *A. maculipennis* s.l*.* is a crucial factor in assessing the potential risk of malaria transmission, the actual risk is indeed determined by the concurrent presence of infected human hosts. Although previous studies have analyzed the distribution of imported malaria cases across non-endemic countries [15], it is the overlap with the distribution of competent vectors that ultimately determines transmission risk [11]. While most of the imported cases in Europe, and particularly in Spain, correspond to *P. falciparum*, most of the local transmission cases in Europe are caused by *P. vivax* and *P. ovale*. This could be due to the higher vector competence of European populations of *A. atroparvus* for these species and the apparent refractoriness of this mosquito species to current variants of *P. falciparum* circulating in Africa [45]. Experiments done using African variants of *P. falciparum* and *A. atroparvus* from Italy, Portugal or Spain, have reported low rates of oocysts development, and no sprorozoites development in the mosquitoes from Italy and Spain [50,51]. Unfortunately, the development of sporozoites was not followed in the study from Portugal, although rates of oocysts development were higher than in the other studies, probably due to the modification of the temperature conditions of the assays [52] and the study from Spain has not been published yet, and consequently the details from the experimental protocols are not available [51]. However, some local transmission cases of *P. falciparum* have been reported in Western Europe, which highlights the need to experimentally determine the competence of autochthonous *Anopheles* species for the transmission of the main *Plasmodium* variants currently circulating worldwide. We incorporated *P. falciparum* into our study because it was one of the original species producing malaria in Spain and owing to the limited scientific evidence supporting the refractoriness of *A. atroparvus*. It should be noted also that imported cases of both *P. ovale* and *P. vivax* are widely distributed throughout Spain´s geography (Figure. S6 See also Figure 3c). These pathogens may cause relapses years after the first infection [53], highlighting the need for appropriate antimalarial treatment of imported cases.

We also need to consider that reporting of imported cases may be biased against segments of the population more reluctant to use medical services, and it may represent an underestimation of the real number of imported cases. In fact, in none of the recent local transmission cases in Spain was possible to identify the imported case that originated the transmission. Improving the reporting system to reduce the presence of incomplete records and improve data quality is imperative given the significant proportion of incomplete registers in RENAVE, especially in relation to the municipality of residence and *Plasmodium* species. The increasing number of imported cases in Spain, except for 2020, probably due to COVID-19 travel restrictions, reflects the improvement of the coverture and communication of cases to RENAVE, and the increase in international travels, and can not be interpreted in itself as an estimator of levels of *Plasmodium* circulation in the countries of origin in the last 15 years [17].

In sum, this study demonstrates the utility of using multidisciplinary approaches to develop high-risk maps to identify areas prone to malaria re-introduction. By combining data on the competent vector distribution, environmental parameters, and imported malaria cases, we have gained insights into the geographical hotspots of transmission risk. This information can be used as a foundation to create comprehensive risk maps that highlight areas with a higher likelihood of malaria transmission. Such maps can be instrumental in guiding public health efforts, including targeted mosquito control measures, enhanced surveillance and monitoring, and the allocation of resources for prevention and treatment. By identifying high-risk areas, we can prioritize interventions and interventions, ultimately working towards the goal of protecting public health.

## Supplementary Material

SupplementaryMaterial
