# Correction

**DOI:** 10.1080/22221751.2024.2357456

**Published:** 2024-05-29

**Article title:** Modeling the spatial risk of malaria through probability distribution of Anopheles maculipennis s.l. and imported cases

**Authors:** Taheri S, González MA, Ruiz-López MJ, Magallanes S, Delacour-Estrella S, Lucientes J, Bueno-Marí R, Martínez-de la Puente J, Bravo-Barriga D, Frontera E, Polina A, Martinez-Barciela Y, Pereira JM, Garrido J, Aranda C, Marzal A, Ruiz-Arrondo I, Oteo JA, Ferraguti M, Gutíerrez-López R, Estrada R, Mirandar MA, Barceló C, Morchón R, Montalvo T, Gangoso L, Goiri F, L. García-Pérez A, Ruiz S, Fernandez-Martinez B, Gómez-Barroso D and Figuerola J

**Journal:** *Emerging Microbes & Infections*

DOI: 10.1080/22221751.2024.2343911

When this article was first published online the colored circles in Figure 1(b) near x1, x2, and x3 was not present, the legend of Figure 2(a) and 4(b) was missing. The Figures have now been corrected.

