# Supplementary material for: Modelling the spatial risk of malaria through probability distribution of Anopheles maculipennis s.l. and imported cases
Source: Emerg Microbes Infect. 2024 Apr 15;13(1):2343911. doi: 10.1080/22221751.2024.2343911 (PMC11073426; doi:10.1080/22221751.2024.2343911)
Supplement: SupplementaryMaterial [file TEMI_A_2343911_SM5536.docx]

**Supplementary Material**

**Modeling the spatial risk of malaria through probability distribution of *Anopheles maculipennis* s.l. and imported cases**

**Methods for entomological data collection and molecular identification of sibling species**

In Spain, 14 *Anopheles* species have been historically reported in the literature, but only three species within the Maculipennis complex are currently present: *A. maculipennis*, *A. atroparvus*, and *A. melanoon* (1,2). Members of the *A. maculipennis* complex are prevalent and well-distributed in Europe and are considered the most important vectors of human malaria in Western Europe (3). The identification of sibling species within this complex is challenging as adults are virtually morphologically indistinguishable, and the existence of incomplete reproductive barriers among members of the complex has been observed (4). Although some clues to separate adults from the sibling species have been proposed based on the scales, shape, and size of wing morphology, further morphometric studies have suggested the ambiguity of these attributes given the existence of variation among different populations (5). Nowadays, recent and modern DNA techniques, mostly based on the Internal Transcribed Spacer 2 (ITS2) region, have been implemented in many countries to determine the presence of the different sibling species of the Maculipennis complex (6).

Larvae and adult stages of *A. maculipennis* s.l. were collected using different methodologies and were grouped into five main categories: BG-Sentinel (19.4%) with lures (CO_2_ or odour attractant), ii) Centre for Disease Control and Prevention (CDC) miniature light traps with or without CO_2_ (51.1%), iii) direct aspiration or sweeping (0.8%), iv) larvae dipping (14.1%) and v) others (14.6%). Anopheline data from inappropriate methods to collect this genus, such as ovitraps and aquatic nets, were not included in the analysis.

To determine the relative prevalence of the different *A. maculipennis* sibling species, 121 specimens were collected from a reasonable number of locations across the Spanish peninsular territory. These specimens were subjected to molecular analysis using a PCR-RFLP protocol (5,7). We amplified the Internal Transcribed Spacer 2 (ITS2) region of ribosomal DNA using the primers 5.8S (5’-ATC ACT CGG CTC GTG GAT CGAT-3’) and 28S (5’-ATG CTT AAA TTT AGG GGG TAG TC-3’) [(Linton et al. 2002)](https://paperpile.com/c/5WK7S6/oqQD). PCR was carried out in 25 μl reaction volumes containing 1X Buffer, 2,5 mM MgCl2, 0,2 mM dNTPs (Bioline, Cincinatti, Ohaio, USA), 0.5 μM of each primer, and 1 Unit of Taq Polymerase (BIOTAQTM DNA polymerase, Bioline, Cincinatti, Ohaio, USA). The thermal cycling conditions were: 94°C for 5 min, followed by 35 cycles of 94°C for 30 sec, 53°C for 30 sec, and 72°C for 30 sec, and a final extension at 72°C for 7 min. The PCR product was further processed with a RFLP protocol that allows distinguishing between *A. atroparvus,* *A. labranchiae*, *A. maculipennis* and *A. melanoon* based on fragment sizes. We first carried out a restriction reaction with the enzyme, HHAI (Fisher Scientific, Waltham, Massachusetts, USA). We added 5 μl of each ITS2 PCR product to 1X restriction enzyme buffer and 1.25 Units of HHA1 enzyme, for a total volume of 20 μl, and incubated the reaction for 3 h at 37°C. We checked the digested fragments on a 2% agarose gel. After this digestion, the RFLP expected sizes were *A. atroparvus* (389 bp fragment), *A. melanoon* (fragments with 108 bp and 135 bp), and *A. labranchiae/A. maculipennis* (300 bp). For those reactions showing fragments around 300 bp, we carried out a new enzymatic reaction using the enzyme HPAII (Fisher Scientific). After this enzymatic digestion, the expected RFLP sizes were *A. labranchiae* (279 bp fragment) and *A. maculipennis* (201 bp fragment). The information on the presence of the different sibling species was completed with previously published information from molecular studies using the same or a similar methodology to identify to species level the *A. maculipennis* complex species (80 extra specimens) (Figure S2).


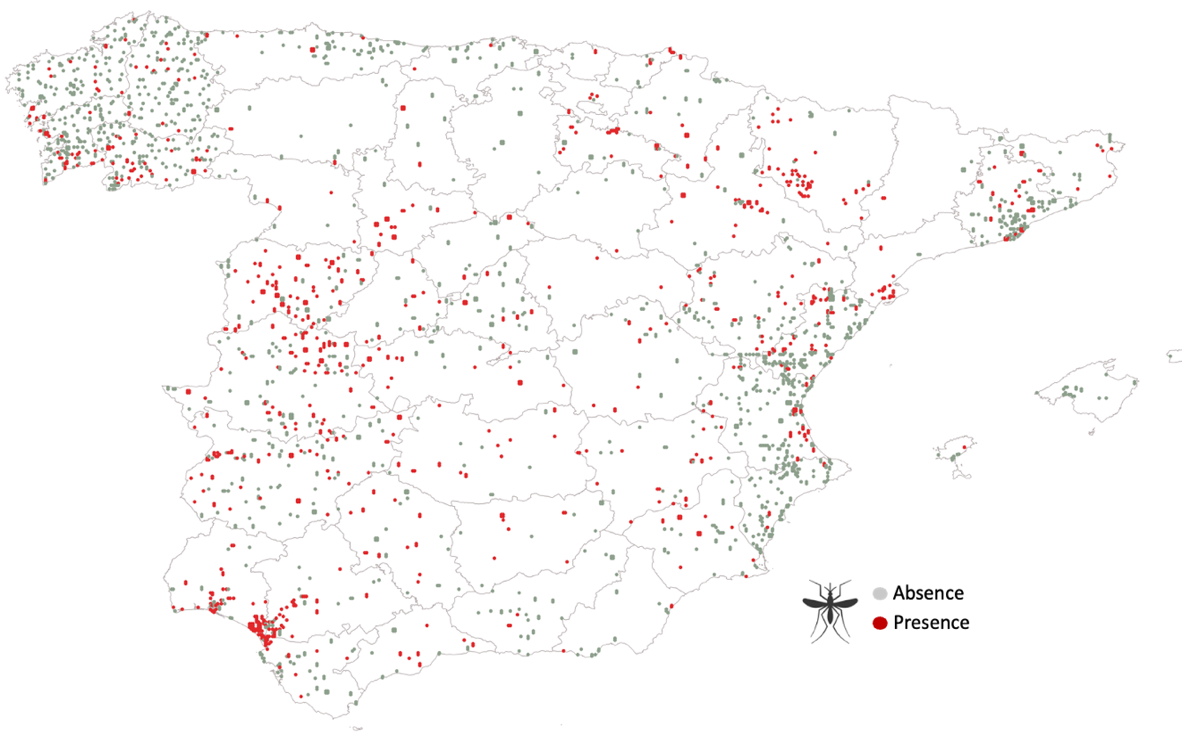


**Figure S1**. Distribution of sample points of A. maculipennis s.l. in Spain in 2 x 2 km grid cells.

**Table S 1**.  Predictors used in the models and what factors of the vectors biology can be affected by them.

| ***No*** | ***Predictor*** | ***Explanation*** |
| --- | --- | --- |
| ***1*** | *Maximum temperature* | *Often related to the abundance, distribution, survival, life cycle of the vectors and extrinsic incubation period of the pathogen** |
| *2* | *Irrigated land* | *Provide breeding sites (larval development)* |
| *3* | *Annual temperature* | *Abundance, distribution, survival, life cycle, extrinsic incubation period** |
| *4* | *Temperature seasonality* | *Abundance, distribution, survival, life cycle, extrinsic incubation period** |
| *5* | *Runoff* | *Modification of mosquito breeding habitats* |
| *6* | *Agriculture* | *Mosquito population dynamics, abundance, breeding sites, host-interactions and land use changes* |
| *7* | *Natural ecosystems* | *Provide breeding sites, refugee and host-interactions* |
| *8* | *Wind* | *Active and passive dispersal and host-seeking activity* |
| *9* | *Urban area* | *Particular conditions unsuitable for Anopheles proliferation* |
| *10* | *Urban green space* | *Provide breeding sites and refuge* |
| *11* | *Water bodies* | *Provide breeding sites (larval development)* |
| *12* | *Precipitation seasonality* | *Provide breeding sites (larval development)* |

*
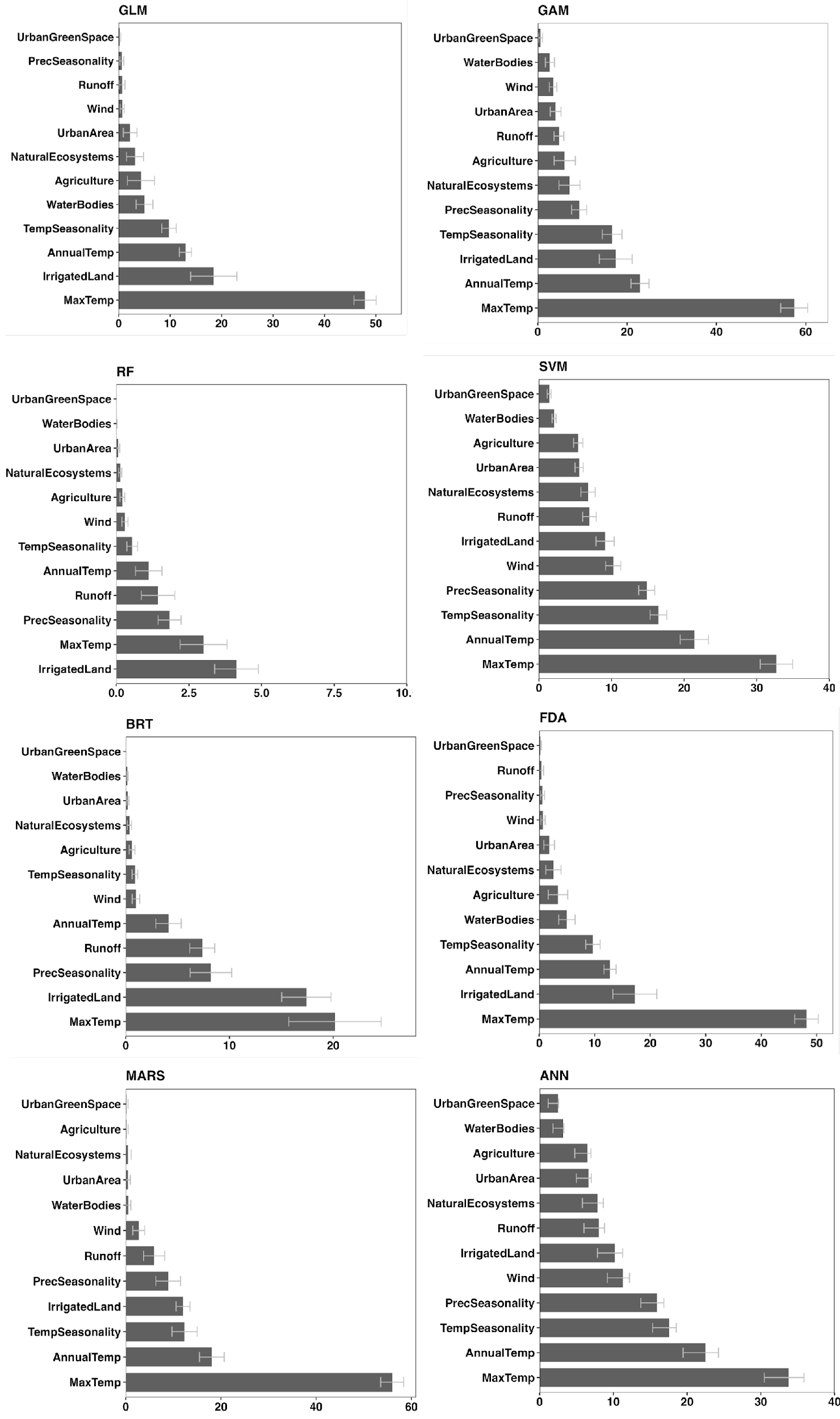
*

**Figure S2**. Variable importance derived from each of the eight different modelling techniques used to build the ensemble model.

**
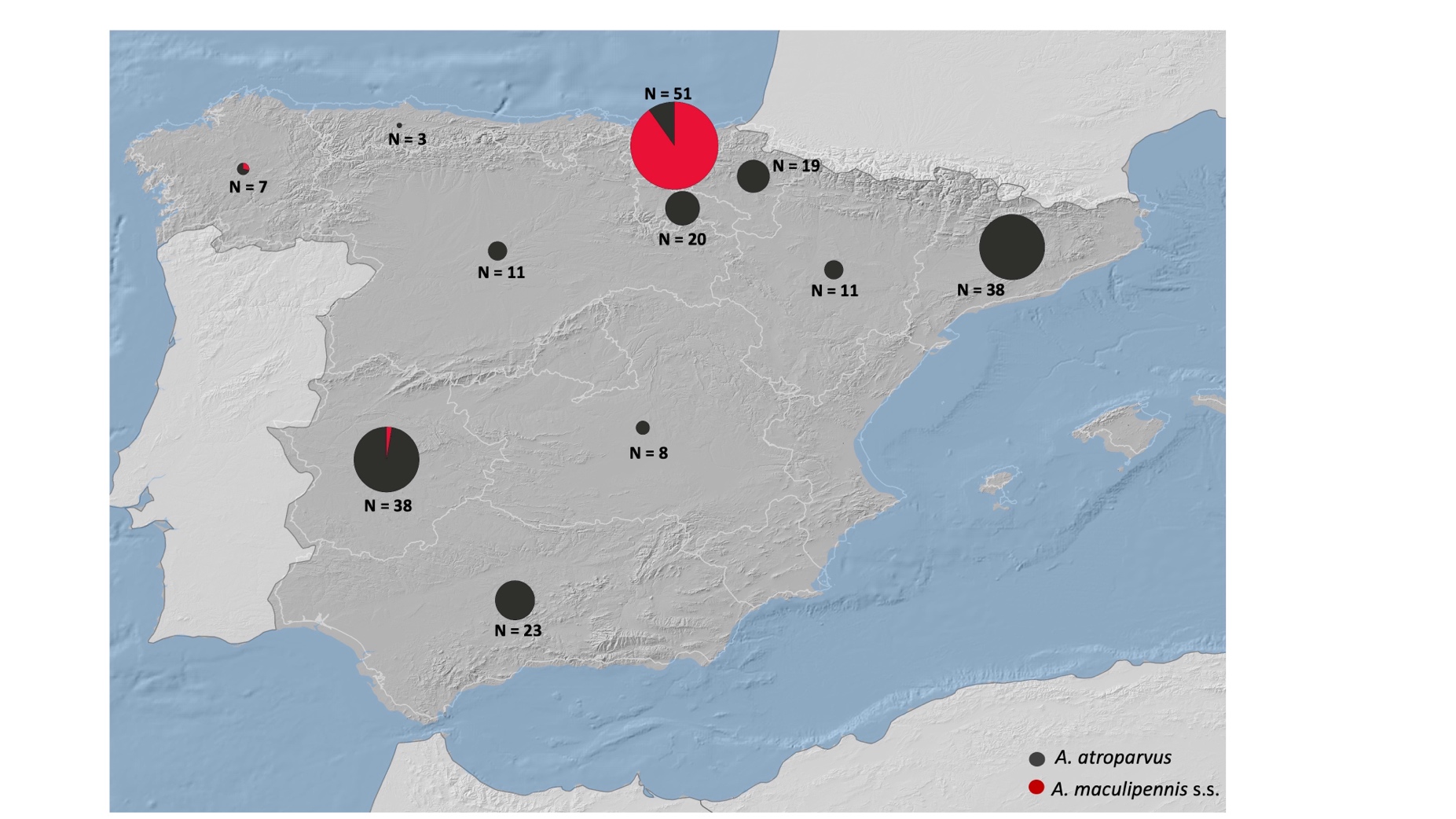
Figure S3**. Molecular analyses of *A.* *maculipennis* s.l. specimens across Spain. The size of the pie charts is proportional to the sample size for each region. The figure combines samples of the individuals examined in the present study (n=121) and other previously published studies (n=108) (8–16).


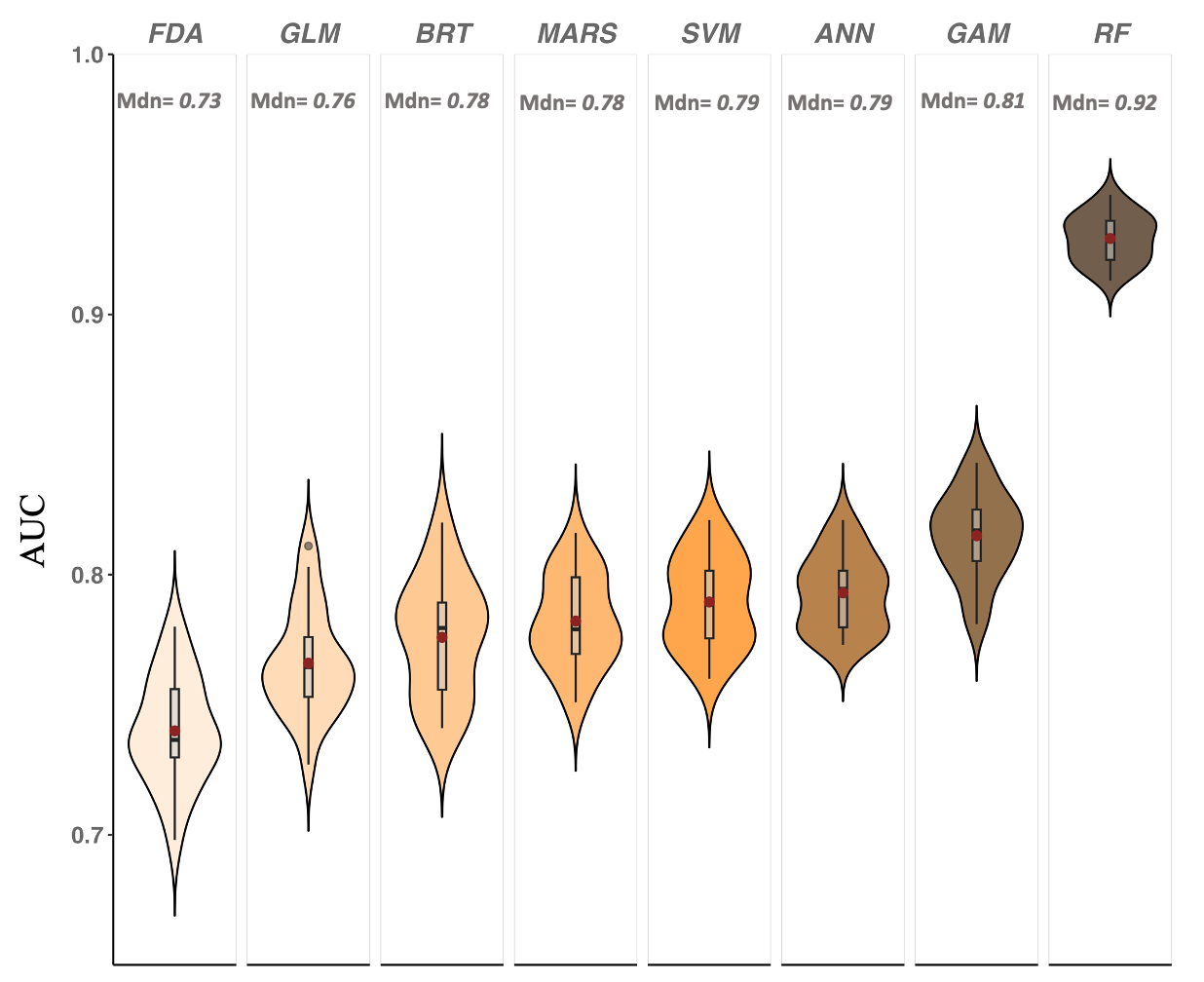


**Figure S4.** Violin plots show the model mean performance (AUC/Area under the ROC curve) for eight modeling techniques, using a test dataset generated from bootstrapping partitioning with 100 replications. The AUC value >80 is considered as very good performance, 0.7 <x<0.8 considered as good model performance, 0.5 <x<0.7 shows the acceptable level of performance, and <0.5 poor performance or as good as random choice. The red dots inside the violin plots indicate the mean performance, and the black line shows the median for each modeling technique. The graphs are color-coded to present a gradient of performance, ranging from lower to higher AUC.


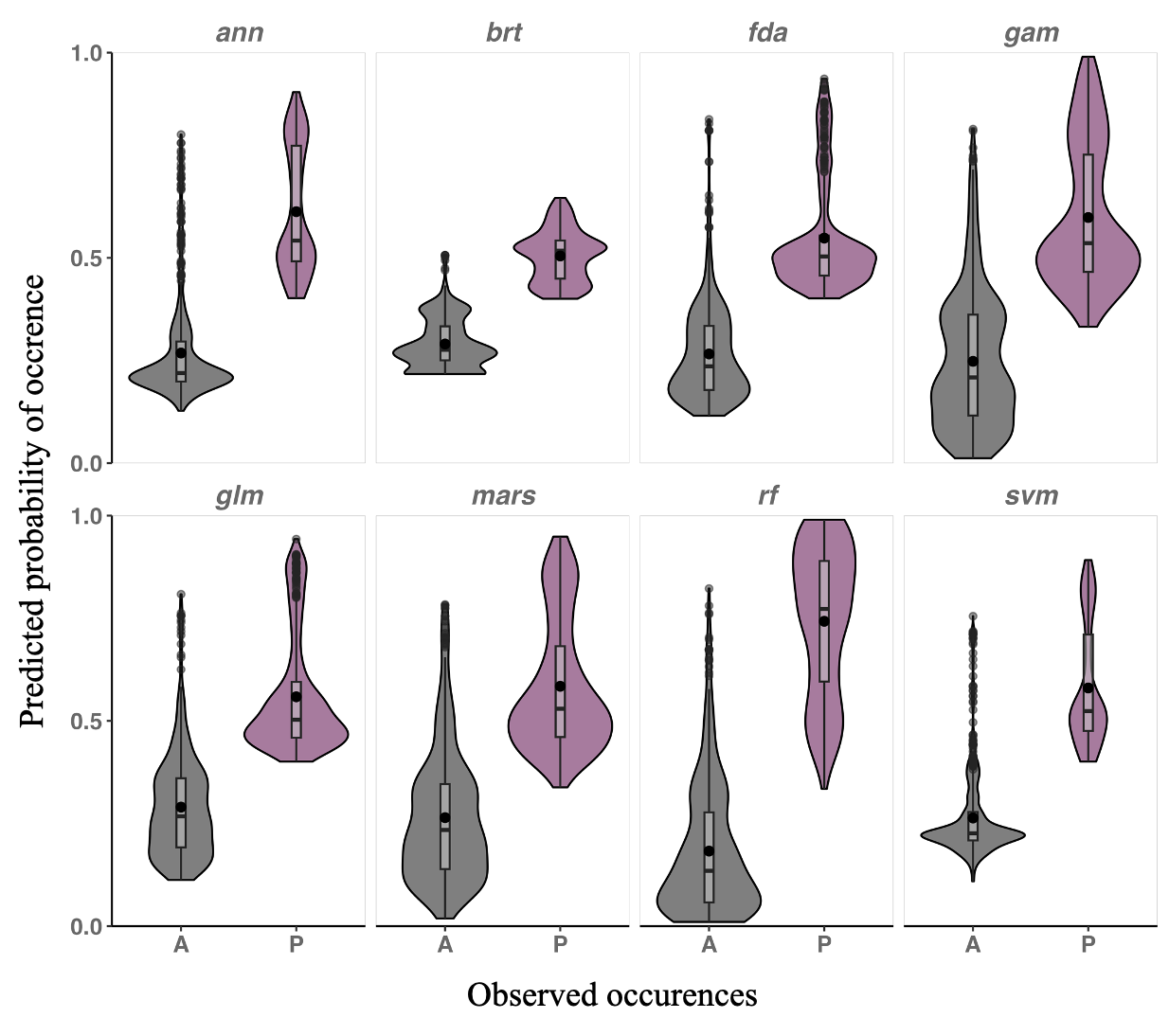


**Figure S5**. Violin plots showing the probability distribution of *A. maculipennis* s.l. in observed presence/absence (P/A) points and indicating how well the model performs in predicting the presence or absence of the species for each modeling technique. The black dots inside the violin plots indicate the mean probability of occurrence, and the black line shows the median. The dark gray violins show the probability of occurrence in absence while purple violin plots display the probability in presence cells.

**Figure S6**. Spatial distribution of malaria parasites across Spain based on imported malaria cases between (2005-2020). **a)** *Plasmodium falciparum*, **b)** *Plasmodium vivax*, **c)** *Plasmodium ovale*, **d)** *Plasmodium malariae* and **e)** not reported.

**Figure S7. Monthly incidence of imported malaria cases from 2005 to 2020 with the reference date being the date of onset of symptoms.** This includes **a)** *Plasmodium falciparum*, **b)** *Plasmodium vivax*, **c)** *Plasmodium ovale*, **d)** *Plasmodium malariae* and **e)** *Not reported*. The time series graphs illustrate the timeline of symptom initiation for these imported malaria cases. The monthly peak of each Plasmodium species and not reported cases is marked by colored dots on the corresponding graph.

**Figure S8.** The probability distribution of *A. maculipennis* s.l*.* and distribution of imported malaria cases overlapped with the most recent instances of local malaria transmission in Spain.


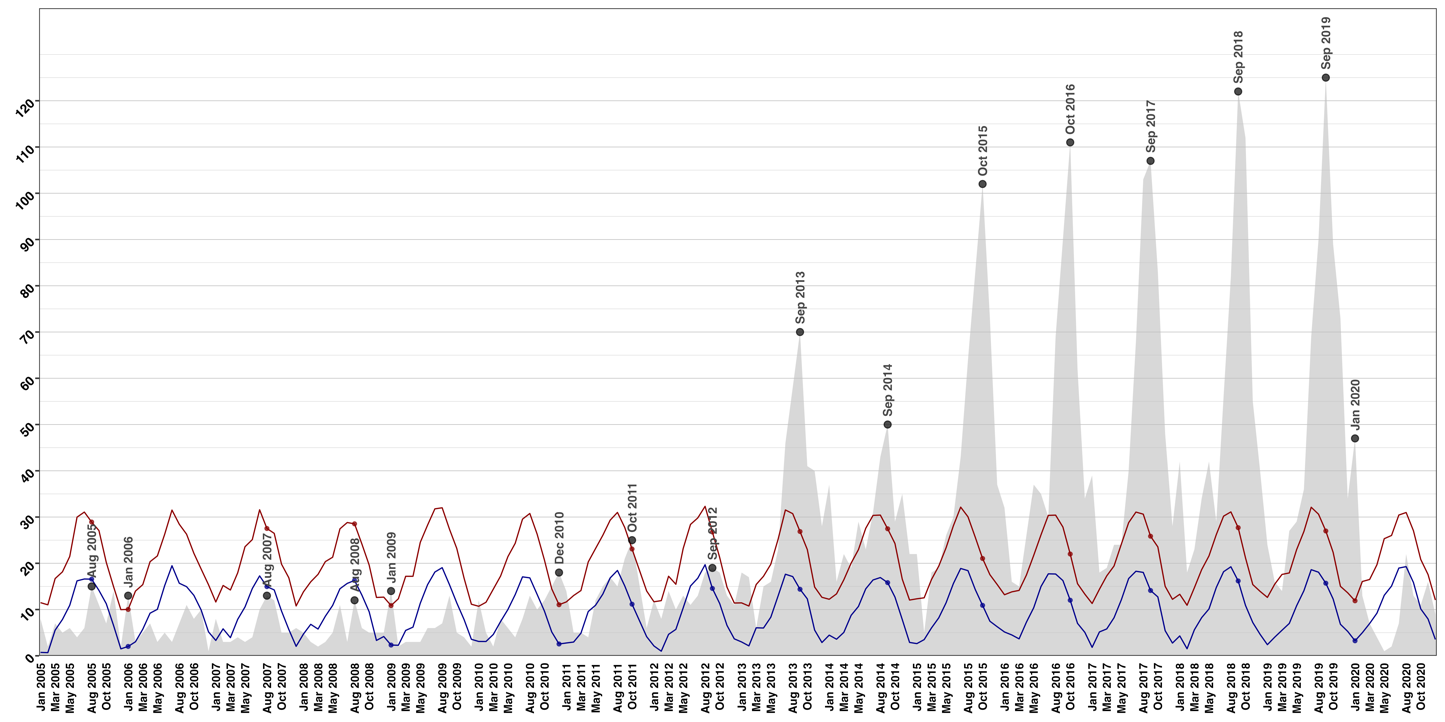


**Figure S9.** The time series plot depicts imported malaria cases' initiation of symptoms date (grey area) alongside the corresponding minimum (blue) and maximum (red) temperatures between 2005 and 2020 (y-axis). Peaks of arrival are represented by black dots on the cases graph, while blue and red dots on the temperature graphs indicate the minimum and maximum temperatures respectively at the time of symptoms initiation during these arrivals.

**References**

1. Eritja R, Aranda C, Padrós J, Goula M, Lucientes J, Escosa R, et al. An annotated checklist and bibliography of the mosquitoes of Spain (Diptera: Culicidae). European Mosquito Bulletin. 2000;(No. 8):10–8.

2. Marí RB, Bernués Bañeres A, Bueno-Marí R, Bernués-Bañeres A, Jiménez-Peydró R. Journal of the European Mosquito Control Association ISSN 1460-6127. European Mosquito Bulletin. 2012;30:91–126.

3. Bertola M, Mazzucato M, Pombi M, Montarsi F. Updated occurrence and bionomics of potential malaria vectors in Europe: a systematic review (2000-2021). Parasit Vectors. 2022 Dec 1;15(1).

4. Linton YM, Smith L, Koliopoulos G, Samanidou-Voyadjoglou A, Zounos AK, Harbach RE. Morphological and molecular characterization of Anopheles (Anopheles) maculipennis Meigen, type species of the genus and nominotypical member of the Maculipennis Complex. Syst Entomol. 2003 Jan 1;28(1):39–56.

5. Vicente JL, Sousa CA, Alten B, Caglar SS, Falcutá E, Latorre JM, et al. Genetic and phenotypic variation of the malaria vector Anopheles atroparvus in southern Europe. Malar J. 2011 Jan 11;10(1):1–9.

6. Kavran M, Zgomba M, Weitzel T, Petric D, Manz C, Becker N. Distribution of Anopheles daciae and other Anopheles maculipennis complex species in Serbia. Parasitol Res. 2018 Oct 1;117(10):3277–87.

7. Collins FH, Paskewitz SM. A review of the use of ribosomal DNA (rDNA) to differentiate among cryptic Anopheles species. Insect Mol Biol. 1996 Feb 1;5(1):1–9.

8. González MA, Goiri F, Cevidanes A, Hernández-Triana LM, Barandika JF, García-Pérez AL. Mosquito community composition in two major stopover aquatic ecosystems used by migratory birds in northern Spain. Med Vet Entomol. 2023 Sep 1;37(3):616–29.

9. González MA, Goiri F, Barandika JF, García-Pérez AL. Culicoides biting midges and mosquito fauna at three dog and cat shelters in rural and periurban areas in Northern Spain. Med Vet Entomol. 2021 Mar 1;35(1):79–87.

10. Bravo-Barriga D, Gomes B, Almeida APG, Serrano-Aguilera FJ, Pérez-Martín JE, Calero-Bernal R, et al. The mosquito fauna of the western region of Spain with emphasis on ecological factors and the characterization of Culex pipiens forms. J Vector Ecol. 2017 Jun 1;42(1):136–47.

11. Ruiz-Arrondo I, Hernández-Triana LM, Nikolova NI, Fooks AR, Oteo JA. Integrated Approaches in Support of Taxonomic Identification of Mosquitoes (Diptera: Culicidae) in Vector Surveillance in Spain. Vector Borne Zoonotic Dis. 2020 Nov 1;20(11):831–42.

12. González MA, Cevidanes A, Goiri F, Barandika JF, García-Pérez AL. Diversity and distribution of larval habitats of mosquitoes (Diptera: Culicidae) in northern Spain: from urban to natural areas. J Vector Ecol. 2021 Nov 8;46(2).

13. González MA, Goiri F, Barandika JF, García-Pérez AL. Culicoides biting midges and mosquito fauna at three dog and cat shelters in rural and periurban areas in Northern Spain. Med Vet Entomol. 2021 Mar 1;35(1):79–87.

14. Bargues MD, Latorre JM, Morchon R, Simon F, Escosa R, Aranda C, et al. rDNA Sequences of Anopheles Species from the Iberian Peninsula and an Evaluation of the 18S rRNA Gene as Phylogenetic Marker in Anophelinae. J Med Entomol. 2006 May 1;43(3):508–17.

15. Proft J, Maier WA, Kampen H. Identification of six sibling species of the Anopheles maculipennis complex (Diptera: Culicidae) by a polymerase chain reaction assay. Parasitol Res. 1999;85(10):837–43.

16. Birnberg L, Aranda C, Talavera S, Núñez AI, Escosa R, Busquets N. Laboratory colonization and maintenance of Anopheles atroparvus from the Ebro Delta, Spain. Parasit Vectors. 2020 Aug 3;13(1):1–5.

17. Marchant P, Eling W, Van Gemert GJ, Leake CJ, Curtis CF. Could British mosquitoes transmit falciparum malaria? Parasitology Today. 1998 Sep 1;14(9):344–5.
